# Rehabilitation for revision total knee replacement: survey of current service provision and systematic review

**DOI:** 10.1186/s12891-023-06196-1

**Published:** 2023-02-03

**Authors:** Ifrah Omar, Setor K. Kunutsor, Wendy Bertram, Andrew J. Moore, Ashley W. Blom, Erik Lenguerrand, Michael R. Whitehouse, Vikki Wylde

**Affiliations:** 1 Musculoskeletal Research Unit, Bristol Medical School, University of Bristol, Learning and Research Building, Southmead Hospital, Bristol, BS10 5NB UK; 2 Southmead Hospital, North Bristol NHS Trust, Bristol, UK; 3 Bristol Biomedical Research Centre, University Hospitals Bristol and Weston NHS Foundation Trust and the University of Bristol, Bristol, UK

**Keywords:** Revision total knee replacement, Rehabilitation, Survey, Systematic review

## Abstract

**Background:**

Revision total knee replacement (TKR) is a major operation with a long recovery period and many patients report suboptimal outcomes. Rehabilitation has the potential to improve outcomes. The aim of this study was to understand current provision of rehabilitation for revision TKR in England and evaluate the existing evidence.

**Methods:**

*Phase 1*: An online national survey of education and rehabilitation provision for patients receiving revision TKR was completed by physiotherapy staff at 22 hospitals across England that were high volume for revision TKR (response rate of 34%).

*Phase 2*: Systematic review to identify studies evaluating rehabilitation programmes for revision joint replacement. Searches were conducted in MEDLINE, EMBASE, PsycINFO, CINAHL, and Cochrane databases from inception to 15^th^ June 2022. Randomised controlled trials (RCTs) and observational studies that evaluated post-operative rehabilitation for adults undergoing revision joint replacement were included. Screening, data extraction and quality assessment was undertaken by two reviewers.

**Results:**

*Phase 1*: Pre-operative education which aimed to prepare patients for surgery and recovery was provided in most hospitals, predominately involving a single session delivered by a multidisciplinary team. Inpatient physiotherapy commonly commenced on post-operative day 1 and was provided twice daily, with most hospitals also providing occupational therapy. Rehabilitation was often provided in the first four weeks after hospital discharge, either in an outpatient, community or home setting. In most hospitals, the education and rehabilitation provided to patients receiving revision TKR was the same as that provided to patients undergoing primary TKR.

*Phase 2*: Of the 1,445 articles identified, three retrospective cohort studies based on hospital records review were included. The studies evaluated intensive inpatient rehabilitation programmes, consisting of 2–3 h of daily group or individual physiotherapy, with additional occupational therapy in one study. All three studies reported improvement in functional outcomes for patients undergoing rehabilitation after revision TKR. All studies were limited by their retrospective design, short duration of follow-up and lack of sample size calculation. No RCTs evaluating effectiveness of rehabilitation for revision TKR were identified.

**Conclusion:**

This study identified the need for future research to develop and evaluate tailored rehabilitation to optimise patient outcomes following revision TKR.

**Supplementary Information:**

The online version contains supplementary material available at 10.1186/s12891-023-06196-1.

## Background

Over 100,000 primary total knee replacements (TKRs) are performed annually in the United Kingdom [1, 2]. The aim of the operation is to improve functional ability and provide relief from chronic pain, most commonly due to osteoarthritis. Implant survivorship is a key concern, with approximately 82% of TKRs lasting 25 years or more [3]. Implants fail for a variety of reasons, including prosthetic wear, aseptic loosening, instability, peri-operative fracture, stiffness and prosthetic joint infection [1]. When an implant fails, revision surgery is required, with implant removal and replacement. Revision surgery can be complex depending on the reason for revision, bone stock, patient age and comorbidities. More complex cases such as prosthetic joint infection typically require multiple surgeries, larger incisions, excision of scar tissue, infected tissue and reconstruction of poor or missing bone stock. Approximately 6000 revision TKRs are performed annually in the United Kingdom [1, 2]. This number is expected to increase in the future due to the predicted increased need for primary TKR [4].

Patients often have high expectations of their outcomes after revision TKR [5], however, revision surgery can have a profound negative impact on patients [6]. Outcomes are often poorer after revision TKR compared with primary TKR: nearly half of patients report severe chronic post-operative pain and 40% reporting limited mobility after revision TKR [7]. The risk of falling is also increased after revision TKR compared with primary TKR [8]. Limited mobility in an ageing population is associated with reduced quality of life, higher mortality, comorbidities, increased hospitalisation and health care costs, adding a substantial burden to healthcare systems [9–11].

Physiotherapy, either provided alone or as part of a multidisciplinary rehabilitation package, can be provided to patients with the goal of facilitating functional recovery and improving outcomes after joint replacement. Current British Orthopaedic Surgery Standards for Trauma and Orthopaedics on revision TKR do not include guidance on rehabilitation provision [12]. Rehabilitation for revision TKR has been identified as an important research priority for patients and clinicians, and features as a top 10 priority in the James Lind Alliance Priority Setting Partnership for revision knee replacement; “What can be done after and/or before revision knee surgery (including physiotherapy and exercise) to optimise the result?” [13].

The aim of our study was to scope current service provision and the existing evidence base for rehabilitation after revision TKR to identify if there is the need for future intervention development work. Specific objectives of the study were to 1.) gather information on current rehabilitation service provision for patients undergoing revision TKR in England using a national survey and 2.) conduct a systematic review of research evaluating rehabilitation interventions and outcomes following revision joint replacement.

## Methods

This was a 2-part study, with phase 1 comprising a national survey of rehabilitation provision for revision TKR and phase 2 comprising a systematic review of existing literature evaluating rehabilitation after revision joint replacement.

### Phase 1: national survey

The 100 highest-volume National Health Service (NHS) hospitals for revision TKR surgery in 2019 were identified from the National Joint Registry in February 2022 [1]. These hospitals performed between 22 and 210 revision TKR procedures in 2019. E-mail addresses for orthopaedic departments or lead rehabilitation specialists were identified from the hospital website or by telephoning the hospital. Contacts were then e-mailed a link to the online survey, with up to two reminders sent to non-responders. The survey, administered using Online Surveys (www.onlinesurveys.ac.uk), consisted of 27 questions which were developed by the research team, informed by previous national surveys of rehabilitation services for primary TKR [14] and revision hip replacement [15]. The survey questions explored pre-operative, in-patient and post-discharge care including pre-operative education, inpatient physiotherapy and occupational therapy, rehabilitation-specific discharge criteria, and provision of post-discharge rehabilitation.

Survey responses were exported into Microsoft Excel. Frequency statistics were used to analyse categorical data. Free-text variables were reviewed and coded into categories by two authors, and a descriptive summary of service provision developed. The project was conducted as a Clinical Effectiveness project, with approval from the North Bristol Trust NHS Quality Governance Team (reference CE97788).

### Phase 2: systematic review

The systematic review protocol was prospectively registered on PROSPERO (CRD42022340099) and reporting follows MOOSE guidance [16]. Although our area of interest was on rehabilitation after revision TKR, we expanded our searches to include all revision joint replacement given the likely similarities in care pathways for these different orthopaedic procedures.

#### Searches

Searches were conducted in MEDLINE, EMBASE, PsycINFO, CINAHL, and Cochrane databases from inception to 15^th^ June 2022. No restrictions were placed on study design or language. The searches combined free and MeSH search terms and combination of key words related to rehabilitation (e.g. physical therapy, rehabilitation, physiotherapy) and revision joint replacement. Reference lists of retrieved articles were manually scanned for all relevant additional studies and review articles. Further details of the search strategies and terms are provided in the online supplementary materials.

#### Inclusion criteria

Studies were included if they evaluated post-operative rehabilitation interventions for adults undergoing revision joint replacement for any indication. All types of revision joint replacement operations were included to maximise the number of studies included in the review. The main outcomes of interest were joint pain and joint function. Eligible study designs included randomised controlled trials and observational studies (prospective and retrospective cohorts, case–control, and nested case–control studies). Studies that included both primary and revision joint replacement patients were eligible for inclusion if they reported results separately for patients undergoing revision joint replacement. Conference abstracts and theses were excluded.

#### Screening

After removal of duplicates in Endnote, study records identified in the searches were imported into Excel for screening. Titles and abstracts were screened to remove clearly irrelevant articles. Detailed screening of potentially relevant articles was then conducted independently by two reviewers (IO and VW) to identify eligible studies for inclusion in the review.

#### Data extraction

Data extraction was conducted by one reviewer and checked by a second reviewer. Data were extracted on study design, publication date, geographical location, participant demographics and surgical details, duration of follow-up, sample size, intervention content and timing, outcomes, results and information for assessment of study quality.

#### Assessment of study quality

For studies with a cohort design, it was planned that methodological quality would be assessed using the Methodological Index for Non-Randomised Studies (MINORS) instrument [17]. MINORs is a validated tool for assessing the quality of non-randomised studies and uses an 8-item checklist to score factors contributing to study quality on a 0–2 point scale, with total scores ranging from 0–16 (low to high quality). For studies with a case–control design, it was planned to assess study quality using the Newcastle–Ottawa Scale (NOS) tool. For randomised controlled trials, quality was planned to be assessed using the Cochrane Collaboration’s risk of bias tool. Methodological quality of included studies was assessed by two reviewers.

#### Data analysis

At protocol stage, meta-analysis was planned if two or more studies were identified with similar rehabilitation programmes and appropriate outcome data. Risk estimates (risk ratios for cohort studies and odds ratios for case–control) and/or mean differences would be used as the common measure of association across studies. Risk estimates would be calculated for studies that reported raw counts. When reported risk estimates could not be calculated, we planned to obtain the relevant estimates through correspondence with the study authors. The inverse variance weighted method would be used to combine summary measures using random-effects models to minimise the effect of between-study heterogeneity. Heterogeneity would be assessed using the I^2^ statistic.

A meta-analysis was not possible due to the limited number of studies and therefore a narrative synthesis was conducted, with the findings of each study summarised in tables and described in a narrative format.

## Results

### Phase 1: national survey

#### Participants

Of the 100 highest-volume NHS hospitals for revision TKR, e-mail addresses were identified for 65 hospitals. An invitation e-mail containing the link to the online survey was sent to a named contact or department e-mail address at the 65 hospitals in March 2022. Of these, 24 physiotherapy staff at 22 hospitals (34%) completed the survey. Respondents were based in NHS hospitals across England, including the South East (10), South West (6), North East (3), North West (1), East Midlands (1) and West Midlands (1).

#### Pre-operative education

Pre-operative education was provided at 73% (*n* = 16) of the responding hospitals and details are provided in Table 1. The pre-operative education provided to patients undergoing revision TKR patients was the same as that provided to patients undergoing primary TKR patients in half of the hospitals. Education was most commonly provided in a single session, within the setting of the pre-operative assessment clinic or knee class/school. Delivery was by nurses, physiotherapists, occupational therapists and orthopaedic surgeons, and in 13 hospitals delivery involved more than one type of healthcare professional. Various formats were used for education provision, with written information being the most common. The content of the education most frequently covered information about the operation, hospital stay and recovery, and advice on pain management and exercise. Only three hospitals provided aids or equipment, these included crutches, toilet equipment, chair/bed raises and dressing aids.

**Table 1:** Pre-operative education for patients undergoing revision TKR

Survey topic	Number of hospitals (*n* = 22)
Pre-operative education provided	
Yes	16
No	3
Unknown	3
Setting^a^	
Pre-operative assessment clinic	11
Knee class/school	7
Outpatient	2
Written documents	2
Telephone	1
Video	1
Number of sessions	
1 session	14
Unknown	2
Healthcare professionals^a^	
Nurse	13
Physiotherapist	11
Occupational therapist	6
Orthopaedic surgeon	6
Rehabilitation assistant	1
Mode of provision^a^	
Written information	14
Talks/presentations	8
Videos/website/app	7
Content^a^	
Information about the operation	15
Information about the hospital stay	15
Information about recovery	15
Pain management	14
Exercise	14
Smoking/alcohol cessation	5
Weight loss	5
Use of equipment/aids	9
Provision of aids and/or specific equipment	
Provided	3
Not provided	10
Unknown	3
Education provision same as primary TKR	
Yes	8
No	1
Unknown	7

^a^Respondents could provide more than one response

#### Post-operative inpatient rehabilitation

Details of post-operative inpatient rehabilitation are provided in Table 2. The inpatient care provided to patients undergoing revision TKR patients was the same as that provided to patients undergoing primary TKR patients in most hospitals. Inpatient physiotherapy commenced on the day of surgery or post-operative day one and was provided once or twice daily, with half of the hospitals also providing occupational therapy. Criteria for hospital discharge were that patients were safe with walking aids, safe with climbing or descending stairs and had adequate social support. Some hospitals had precautions, these were partial weightbearing, avoiding twisting, no kneeling and avoiding fixed flexion when sleeping.

**Table 2:** Post-operative inpatient rehabilitation for patients with revision TKR

Survey topic	Number of hospitals (*n* = 22)
Commencement of physiotherapy	
Day of surgery	8
Post-operative day 1	14
Frequency of physiotherapy	
Once per day	8
Twice per day	12
Unknown	2
Occupational therapy	
Provided	11
Not provided	6
Unknown	5
Criteria for discharge^a^	
Safe with walking aids	22
Safe with climbing or descending stairs	22
Adequate social support	21
Appropriate range of motion	2
Precautions^a^	
None	8
Partial weightbearing	4
Avoid twisting	3
No kneeling	2
Sleeping position	1
Unknown	5
Precautions same as primary TKR	
Yes	13
No	1
Unknown	8
Inpatient care same as primary TKR	
Yes	19
No	1
Unknown	2

^a^Respondents could provide more than one response

#### Post-discharge rehabilitation

All hospitals provided post-discharge rehabilitation, either to all patients or to patients with poor mobility or range of motion (Table 3). The post-discharge rehabilitation provided to revision TKR patients was the same as that provided to primary TKR patients in most hospitals. Rehabilitation was commonly provided within the first four weeks of hospital discharge, either in hospital outpatient departments, the community or patient’s homes. Most hospitals provided rehabilitation in more than one format, including as individual sessions, telephone/videocall, written information, unsupervised home exercises, group-based classes and home visits. The number of sessions varied, with 2–6 sessions being the most common. Most responding hospitals provided more than one treatment modality, these included functional and joint-specific exercises, advice, ice/heat, hydrotherapy and manual therapy.

**Table 3:** Rehabilitation following hospital discharge for patients with revision TKR

**Survey topic**	**Number of hospitals (*n* = 22)**
Provision of post-discharge rehabilitation	
Provided to all patients	16
Provided to patients who meet specific criteria	6
Timing of rehabilitation provision	
Within 2 weeks	8
2–4 weeks	9
As soon as possible/when appropriate	2
Unknown	3
Location^a^	
Hospital outpatients	20
Community	12
Home	13
Format^a^	
Individual session	21
Telephone/videocall	15
Written information	13
Unsupervised home exercise programme	13
Group-based class	12
Home visit	11
Unknown	1
Number of sessions	
< 2	1
2–4	4
5–6	7
> 6	1
Unknown	9
Treatment modalities	
Functional exercises (including gait re-education)	19
Advice	18
Specific joint exercise (strengthening/stretches/ROM)	18
Ice/Heat	9
Hydrotherapy	9
Manual therapy (including soft tissue techniques)	7
Pain management (including CBT)	2
Occupational Therapy	2
Electrotherapy	1
Unknown	3
Post-discharge rehabilitation provision same as primary TKR	
Yes	20
Unknown	2

^a^Respondents could provide more than one response

### Phase 2: systematic review

#### Study characteristics

**Fig. 1:**
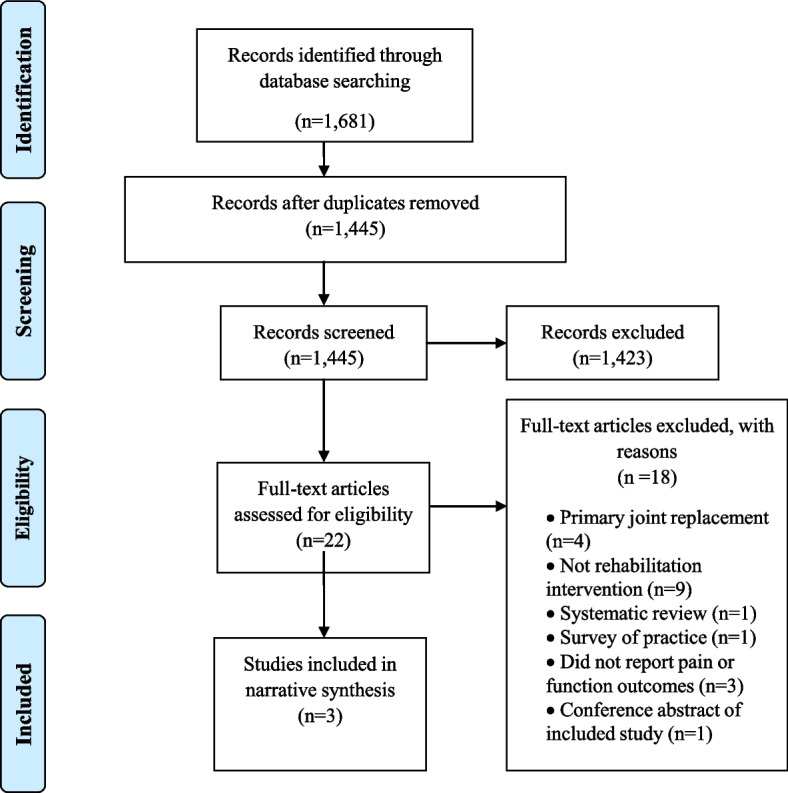
PRISMA flow diagram

After removal of duplicates, searches identified 1,445 study records. After initial screening, 22 articles were identified as potentially relevant and screened in detail. Of these, three met the inclusion criteria and were included in the review [18–20] (Fig. 1). Summaries of these studies are provided in Table 4. All three studies were retrospective studies and no randomised controlled trials evaluating the effectiveness of rehabilitation for revision TKR were identified.

**Table 4:** Summary of study characteristics

**Author** **Country** **Study dates** **Setting** **Study design**	**Inclusion** **Number recruited** **Mean age** **% female**	**Intervention description**	**Follow up** **Relevant outcomes** **Losses to follow up** **MINOR study quality score** **Key results**
Larsen et al., 2002 [20] Denmark February 2017-June 2018 Inpatient rehabilitation hospital Retrospective hospital register-based cohort study	Patients aged 40–80 years with complications after primary or revision TKR 51 revision TKR patients 62 years 59% female	3 weeks of group-based and personalized rehabilitation supervised by physiotherapists. Groups consisted of 11 participants, with 2–4 sessions daily, each lasting 30–50 min. Sessions targeted neuromuscular function, postural control, flexibility, muscle strength, cardiovascular function, gait retraining and education. Exercises included pelvic lifts, sit-ups, sliding exercises, lunges, rubber band exercises, and functional movements like chair stands and stair climbing. Exercises were adjusted based on individual level	Hospital discharge KOOS, pain NRS, 6-min walk test, stair climb test, knee range of motion 27% (from whole cohort of primary and revision TKR) MINORS score of 11 Patients reported statistically significant improvements in all outcome measures
Vincent et al., 2006 [19] USA January 2002-March 2005 Inpatient rehabilitation hospital Retrospective cohort study using computerized medical records	Patients with primary or revision TKR 138 revision TKR patients Mean age not reported 70% (primary and revision TKR patients)	3 h of supervised therapy daily from both physical and occupational therapists (2 sessions per day) until discharge. Sessions focused on flexibility, range of motion, activities of daily living, gait, balance and proprioception. Patients also used a continuous passive motion machine for 6–8 h daily	Hospital discharge FIM score Losses to follow up not reported MINORS score of 7 FIM scores improved significantly from admission to hospital discharge
Walker et al., 2001 [18] USA 1994–1998 Inpatient rehabilitation hospital Retrospective cohort study using computerized medical records and patient charts	Patients with primary or revision THR 39 revision THR patients 74 years 80% female	3 years of daily supervised rehabilitation involving occupational therapy, physical therapy, and standard THA range-of-motion precautions. One hour of the therapy time was in a structured group session	Hospital discharge FIM score Losses to follow up not reported MINORS score of 7 FIM scores improved from admission to hospital discharge

Larsen et al. report a retrospective hospital register-based study of 51 patients with complications after revision TKR admitted to an inpatient rehabilitation hospital in Denmark between 2017 and 2018 [20]. Vincent et al. conducted a retrospective study using computerised medical records of 138 patients admitted to a rehabilitation hospital in the USA after revision TKR between 2002 and 2005 [19]. In the study by Walker et al., the outcomes of 39 patients admitted to a USA inpatient rehabilitation hospital after revision hip replacement between 1994 and 1998 were evaluated from hospital computerised medical records and patient charts [18]. Study quality ratings were between 7–11, with all studies limited by their retrospective design, short duration of follow-up and lack of sample size calculation.

#### Study interventions

All three studies evaluated intensive rehabilitation programmes delivered in an inpatient hospital setting. The rehabilitation programmes consisted of 2–3 h of daily supervised group or individual physiotherapy, with additional occupational therapy in one study [19]. One study specifically reported that the rehabilitation programme was personalised to adjust exercises to patients’ ability, fatigue and pain levels [20]. Larsen et al. and Vincent et al. reported that the rehabilitation programme was multimodal and aimed to improve outcomes such as range of motion, ability to participate in activities of daily living, balance, and gait [19, 20]; the study by Walker and colleagues did not report on the content on the therapy sessions. In the rehabilitation programme reported by Larsen et al., educational sessions were also provided to give participants information about pain management, their prosthesis, exercise, and advice on to how to continue exercising after hospital discharge. In the study by Vincent et al., participants also used a continuous passive motion machine for 6–8 h daily. Rehabilitation was provided throughout the inpatient stay for all studies, this was standardised to three weeks for Larsen et al., an average of 10.5 days in Walker et al.and variable in the study by Vincent et al., dependent on when patients met their functional goals.

#### Outcomes

All three studies evaluated patients on admission and discharge to the rehabilitation programme. All assessed patient-reported outcomes; Larsen et al. used the Knee injury and Osteoarthritis Outcome Score and a pain Numeric Rating Scale and both Vincent et al. and Walker et al. used the Functional Independence Measure. Additional, Larsen and colleagues assessed objective measures of functional ability including the 6-min walk test, stair climb test and knee range of motion. All three studies demonstrated improvements in these outcomes from admission to discharge from the rehabilitation hospital.

## Discussion

This article reports on a national survey and systematic review to understand current service provision in England and the existing evidence-base for physiotherapy after revision TKR. The national survey found that most responding NHS hospitals provided patients undergoing revision TKR with pre-operative education, inpatient rehabilitation and outpatient rehabilitation after hospital discharge. However, the education and rehabilitation provided to patients following revision TKR was the same as that provided to patients undergoing primary TKR, suggesting that neither are tailored to the needs of patients recovering from revision TKR. The systematic review identified an important gap in the existing literature regarding rehabilitation after revision TKR, with only three published studies evaluating patient outcomes after rehabilitation following revision joint replacement. Narrative synthesis suggests that intensive, inpatient rehabilitation programmes have the potential to improve short-term patient outcomes after revision joint replacement. However, the conclusions that can be drawn from the narrative synthesis are very limited as it was based on a small number of retrospective observational studies. Due to the lack of randomised controlled trials, no inferences can be made about the effectiveness of rehabilitation for patients undergoing revision TKR.

There are a number of strengths and limitations of this research that need to be considered when interpreting the findings. Although we obtained a good geographic spread of hospitals in our national survey, the response rate of 34% was lower than previous surveys, despite using established procedures [14, 21]. Although the reasons for this are not known, it could reflect the current pressure on NHS services and staff following the pause in the provision of elective orthopaedic surgery during the Covid-19 pandemic and the subsequent impact on waiting lists. The systematic review involved comprehensive searches conducted on multiple databases, with a broad scope to include all types of revision joint replacement. Despite this, the analysis and conclusionsthat can be drawn were limited by the small number of studies and the lack of randomised controlled trials to provide high quality evidence on the effectiveness of rehabilitation interventions. The rehabilitation programmes were all intensive inpatient programmes, with no studies evaluating home-based or outpatient programmes. However, importantly our systematic review identified an important gap within the research evidence base. These findings add to the limited existing literature on the provision of rehabilitation for patients undergoing revision joint replacement. In 2016, a national survey of care pathways for patients receiving revision surgery for prosthetic joint infection found that there was a lack of tailored physiotherapy and occupational therapy services for patients [21]. The provision of rehabilitation after revision total hip replacement was evaluated in a national survey in 2016, which found there was considerable variation in service provision and a lack of consensus regarding optimal rehabilitation strategies [15]. Our study adds to this body of evidence, finding that physiotherapy provision for patients undergoing revision TKR is variable and the same as that provided to patients undergoing primary TKR.

We identified a paucity of research evaluating the impact of rehabilitation interventions on pain and function following revision TKR. A number of systematic reviews of randomised controlled trials evaluating rehabilitation for primary TKR have been conducted, with the general consensus that physiotherapy is effective at improving short-term outcomes, although optimising format and content require further investigation [22, 23]. In 2016, a systematic review of healthcare needs and support for patients undergoing treatment for prosthetic joint injection or other major adverse occurrences after hip or knee replacement found no studies that evaluated support interventions, highlighting a lack of evidence to guide service provision [24]. Our systematic review demonstrates a continued paucity of high-quality research to guide rehabilitation and optimise patients’ outcomes after revision TKR.

## Conclusions

Our results highlight a paucity of research to guide the provision of evidence-based tailored rehabilitation for patients undergoing revision TKR. Surgical treatment of major adverse occurrences after primary TKR poses a major burden to patients and the NHS. There is a clear and pressing need for co-interventions to optimise outcomes after surgery and support patients through treatment and recovery. Tailored rehabilitation, within a broader integrated care pathway to address the physical, psychological, emotional, and social needs of patients undergoing revision TKR, could support patients through their treatment journey. Development of novel and individualised rehabilitation programmes would benefit from a co-production approach with key stakeholders, including patients, and use of a relevant theoretical framework to support behaviour change. Evaluation of such interventions would need to be within the framework of a multicentre randomised controlled trial, with longer-term follow-up, cost-effectiveness analysis, and assessment of outcomes that are important and meaningful to patients. Understanding how to measure outcomes following revision TKR in a way that is meaningful to patients is a top 10 research priority in the James Lind Priority Setting Partnership for revision knee replacement [13], and further research is needed to develop a core outcome set for revision TKR.

## Supplementary Information

**Additional file 1.**

## Data Availability

The datasets used and/or analysed during the current study are available from the corresponding author on reasonable request.

## Abbreviations

NHS: National Health Service
MINORS: Methodological Index for Non-Randomised Studies
TKR: Total knee replacement
