# Supplementary material for: Rehabilitation for revision total knee replacement: survey of current service provision and systematic review
Source: BMC Musculoskelet Disord. 2023 Feb 3;24:91. doi: 10.1186/s12891-023-06196-1 (PMC9894733; doi:10.1186/s12891-023-06196-1)
Supplement: Supplementary file 1 — Additional file 1. [file 12891_2023_6196_MOESM1_ESM.docx]

**Search strategies**

| **Database: Ovid MEDLINE(R) <1946 to present>**  **Search Strategy:** |
| --- |
| 1 exp Physical Therapy Modalities/ (171608)  2 exp Rehabilitation/ (339978)  3 exp Exercise Therapy/ or Physiotherapy.mp. (80432)  4 Counselling.mp. or exp Counseling/ (71767)  5 Psychological support.mp. (5066)  6 exp Education/ (876382)  7 exp Occupational Health/ (35978)  8 exp Palliative Care/ or Supportive care.mp. (77685)  9 Support pathways.mp. (47)  10 exp Self-Help Groups/ (10600)  11 exp Arthroplasty, Replacement/ or exp Arthroplasty/ (82886)  12 Joint replacement.mp. (8096)  13 exp Joint Prosthesis/ (47625)  14 revision.mp. (96434)  15 1 or 2 or 3 or 4 or 5 or 6 or 7 or 8 or 9 or 10 (1368714)  16 11 or 12 or 13 (108993)  17 14 and 15 and 16 (570)  18 limit 17 to humans (567) |
|  |
| **Database: Embase <1974 to 2022 June 14>**  **Search Strategy:** |
| 1 Physical therapy.mp. or exp physiotherapy/ (109961)  2 exp rehabilitation/ (446784)  3 Exercise therapy.mp. or exp kinesiotherapy/ (92819)  4 Counselling.mp. or exp counseling/ (203879)  5 Psychological support.mp. (8589)  6 exp education/ (1566090)  7 exp occupational health/ (246943)  8 Supportive care.mp. (36089)  9 Support pathways.mp. (68)  10 Self-help group.mp. or exp self help/ (14484)  11 exp arthroplasty/ or exp total arthroplasty/ (93607)  12 Joint replacement.mp. (11890)  13 exp replacement arthroplasty/ (37888)  14 exp joint prosthesis/ (73279)  15 revision.mp. or exp revision arthroplasty/ (127932)  16 1 or 2 or 3 or 4 or 5 or 6 or 7 or 8 or 9 or 10 (2485786)  17 11 or 12 or 13 or 14 (143993)  18 15 and 16 and 17 (1537)  19 limit 18 to (human and exclude medline journals) (203) |
|  |
| **Cochrane library** |
| “Physical therapy” OR Rehabilitation OR Physiotherapy OR “Exercise therapy” OR Counselling OR counselling OR “Psychological support” OR Education OR “Occupational health” OR “Supportive care” OR “Support pathways” OR “Self-help group” in Title Abstract Keyword AND arthroplasty OR “joint replacement” OR “total replacement” OR “total arthroplasty” OR “replacement arthroplasty” OR “joint prosthesis” in Title Abstract Keyword AND Revision in Title Abstract Keyword - in Trials |
|  |
| **Database: APA PsycInfo <1806 to June Week 2 2022>**  **Search Strategy:** |
| 1 exp Physical Therapy/ (3302)  2 exp Rehabilitation/ (52905)  3 Physiotherapy.mp. (2525)  4 exp Exercise/ or Exercise therapy.mp. (31636)  5 exp Counseling/ or Counselling.mp. (88926)  6 exp Social Support/ or Psychological support.mp. (42921)  7 exp Education/ (465743)  8 exp Occupational Health/ (4841)  9 Supportive care.mp. (1750)  10 Support pathways.mp. (27)  11 exp Group Psychotherapy/ or exp Self-Help Techniques/ or Self-help group.mp. (36162)  12 Arthroplasty.mp. (777)  13 Replacement arthroplasty.mp. (4)  14 Joint replacement.mp. (207)  15 Joint prosthesis.mp. (12)  16 revision.mp. (18874)  17 Revision arthroplasty.mp. (3)  18 1 or 2 or 3 or 4 or 5 or 6 or 7 or 8 or 9 or 10 or 11 (687551)  19 12 or 13 or 14 or 15 (876)  20 16 or 17 (18874)  21 18 and 19 and 20 (6)  22 limit 21 to human (6) |
|  |
| **CINHAL** |
| (Physical therapy OR Rehabilitation OR Physiotherapy OR Exercise therapy OR Counselling OR counselling OR Psychological support OR Education OR Occupational health OR Supportive care OR Support pathways OR Self-help group OR self-help techniques OR psychotherapy ) AND ( arthroplasty OR joint replacement OR total replacement OR total arthroplasty OR replacement arthroplasty OR joint prosthesis ) AND ( Revision OR revision arthroplasty) |
